# Effectiveness of an Evidence-Based Multidisciplinary Approach for Patients With Multiple Comorbidities Admitted to a Quarantine Facility in a Tertiary Hospital During the COVID-19 Pandemic

**DOI:** 10.7759/cureus.88237

**Published:** 2025-07-18

**Authors:** Dialdin Ibrahim, Brijesh Sathian, Arnab Ghosh, Shafi Hashmath Ulla Khan, Hanadi Al Hamad

**Affiliations:** 1 Department of Geriatrics and Long-Term Care, Rumailah Hospital, Hamad Medical Corporation, Doha, QAT; 2 Department of Pathology, Manipal Tata Medical College, Jamshedpur, IND

**Keywords:** chronic diseases, comorbidities, covid-19, emergency management, high-risk patients, infection control, multidisciplinary approach, patient outcomes, qatar, quarantine

## Abstract

Background: The coronavirus disease (COVID-19) pandemic necessitates quarantine measures for travelers, including those with multiple comorbidities. This study examined the effectiveness of a multidisciplinary approach in managing high-risk patients admitted to a quarantine facility in Qatar during the pandemic.

Methods: A retrospective analysis was conducted on 62 patients admitted to the quarantine facility of the Rumailah Hospital from March 1 to October 25, 2020. Data on demographics, comorbidities, clinical management, infection rates, and outcomes were collected and analysed.

Results: The cohort included 62 (100.0%) patients: 22 (35.5%) male individuals and 40 (64.5%) female individuals, with 25 (40.3%) Qatari nationals. Common comorbidities were hypertension 51 (82.3%), diabetes 49 (79.0%), and chronic kidney disease 26 (41.9%). In-hospital morbidity occurred in 4 (6.5%) patients, with no significant sex or nationality differences. Post-discharge, 15 (60.0%) Qatari patients were transferred to long-term care compared to 8 (21.6%) non-Qatari patients, while 22 (59.4%) non-Qatari patients were discharged home compared to 8 (32.0%) Qatari patients. All patients (62 (100.0%))received comprehensive geriatric assessments and multidisciplinary care. No in-hospital mortality was observed.

Conclusion: The multidisciplinary approach effectively managed elderly patients with multiple comorbidities during quarantine, achieving low morbidity and no in-hospital mortality. Comprehensive, patient-centered care mitigated severe infection risks in vulnerable populations during a public health emergency.

## Introduction

The World Health Organization (WHO) declared the outbreak of a novel coronavirus a global public health emergency on January 31, 2020, marking the onset of Coronavirus Disease 2019 (COVID-19). In response, many countries implemented quarantine measures to curb the spread. For instance, on March 1, 2020, Qatar reported its first case-a 36-year-old woman returning from Iran-and promptly enforced a 14-day mandatory quarantine for travelers and close contacts of infected individuals [1]. This included retesting before discharge to identify symptomatic or delayed-onset cases.

Qatari citizens and residents abroad for medical reasons were repatriated and quarantined under national infection control protocols. Recognizing that elderly individuals and those with comorbidities are particularly vulnerable to severe COVID-19, health officials admitted this group to Rumailah Hospital's quarantine unit. Here, a multidisciplinary team delivered comprehensive care, addressing both physical and psychological needs.

To better understand the psychological toll of quarantine, evidence from other regions is instructive. For example, a nationwide analysis in China found that a significant proportion of COVID-19 patients suffered from comorbidities, with those affected experiencing substantially poorer clinical outcomes, highlighting additional stressors on both mental and physical health during the pandemic [2]. Notably, younger individuals exhibited higher rates of generalized anxiety disorder (GAD) and depression compared to older adults [3]. Similarly, Haddad et al. explored how quarantine stresses influenced eating behaviors during the pandemic, comparing clinic attendees with the general population [4]. These findings demonstrate the importance of integrated psychological support in quarantine settings.

Elderly patients with comorbidities face amplified risks due to reduced organ function and impaired responses to hypoxia and inflammation. SARS-CoV-2 primarily targets the lungs but also damages the immune system, liver, blood vessels, and other organs, with evidence of viral particles in liver cells and associated inflammation. Consequently, multidisciplinary teams (MDTs) are essential for personalized treatment, accurate prognosis, and mortality reduction.

The MDT approach adheres to three principles: interdisciplinary collaboration, individualized plans, and proactive monitoring. Teams typically include specialists in critical care, infectious diseases, respiratory medicine, cardiology, nephrology, endocrinology, gastroenterology, neurology, nutrition, rehabilitation, psychiatry, and nursing. Treatment encompasses antiviral and anti-inflammatory therapies, nutritional support, psychological interventions, and rehabilitation. For severe cases, round-the-clock ward rounds enable early detection of deterioration.

Yang et al. showed that their approach worked well by treating 77 patients with severe or critical COVID-19 (average age 63.8 years) using a team-based, comprehensive strategy that lowered death rates and increased recovery rates [5].

In Qatar's Rumailah Hospital protocol, nasal and pharyngeal swabs were collected upon arrival at the airport and tested via reverse transcriptase polymerase chain reaction (RT-PCR) (Thermo Fisher Scientific, Waltham, MA, USA). Symptomatic positives were hospitalized in dedicated facilities, while asymptomatic negatives underwent 14-day isolation. Monitoring included symptom checks (e.g., fever >38°C, cough, myalgia, dyspnea), repeat testing for emerging symptoms, and routine labs like complete blood counts, liver function tests, and chest X-rays.

Comparative studies, such as the one by Dursun et al. on Turkish repatriates, found that severe disease was linked to being 65 or older, having a high fever, muscle pain, and low lymphocyte levels using a specific type of statistical analysis [6]. Building on these insights, our study examined clinical management in Qatar's quarantine unit.

The primary objective of this study was to evaluate the clinical care, infection rates, and characteristics of elderly patients with multiple comorbidities admitted to a quarantine facility at Rumailah Hospital during the COVID-19 pandemic, assessing the effectiveness of a multidisciplinary team (MDT) approach. Secondary objectives included determining the prevalence of severe infections, in-hospital morbidity and mortality, post-discharge disposition, and polypharmacy in this cohort. We hypothesized that the MDT approach would be associated with lower infection rates and improved outcomes in this vulnerable population.

## Materials and methods

Study design and setting

This retrospective study was conducted at the quarantine facility of Rumailah Hospital, a tertiary care center in Qatar specializing in geriatrics and long-term care, during the COVID-19 pandemic. Rumailah Hospital was selected as the study site because it is a leading facility under Hamad Medical Corporation dedicated to elderly and vulnerable populations, with specialized units like the Enaya long-term care center designed for patients requiring ongoing monitoring and multidisciplinary support. As part of Qatar's public health plan to protect vulnerable groups, the hospital was chosen to quarantine elderly people (aged 60 and older) with several health issues who are at higher risk of serious illness from SARS-CoV-2 infection, enabling focused care in a safe environment while reducing infection risks. The study period extended from March 1, 2020, to October 25, 2020.

Participants

All patients admitted to the quarantine facility during the study period were considered for inclusion. Inclusion criteria comprised adults aged 60 years or older who arrived at the facility from international locations or local transfers as part of Qatar’s national quarantine measures. Patients with incomplete or missing data in the electronic medical records were excluded. Following the application of these criteria to the electronic medical records, a total of 62 patients were selected for the study, representing the complete eligible cohort without any further sampling or subgroup selection. No imputation methods or sensitivity analyses for missing data were required or performed.

Variables

The primary variables included demographic information (age, gender, and nationality), comorbidities (hypertension, diabetes mellitus, chronic kidney disease, ischemic heart disease, congestive heart failure, stroke, peripheral vascular disease, thyroid disease, and dementia), and clinical parameters such as vital signs, laboratory results, and COVID-19 diagnostic tests (RT-PCR; Thermo Fisher Scientific, Waltham, MA, USA). Outcomes of interest included in-hospital morbidity, mortality, and post-discharge disposition (home, long-term care, or other facilities).

Data collection

Data were collected retrospectively from the hospital's electronic medical records system. Variables captured included patient demographics, comorbidity profiles, COVID-19 test results, and clinical management details. The dataset also incorporated information on interventions by MDTs, including comprehensive geriatric assessments, dietary support, psychological interventions, and rehabilitation services.

Multidisciplinary care

All patients received care under a multidisciplinary approach. The MDT included specialists in geriatrics, internal medicine, infectious diseases, clinical pharmacy, dietetics, physiotherapy, occupational therapy, and respiratory therapy. Personalized care plans were developed for each patient, incorporating individual clinical needs.

Statistical analysis

Descriptive statistics were used to summarize baseline characteristics, comorbidity prevalence, and clinical outcomes. Categorical variables were expressed as frequencies and percentages. Data analysis was performed using R statistical software (version 3.4.1) (R Foundation for Statistical Computing, Vienna, Austria, https://www.R-project.org).

Ethical considerations

Ethical approval for the study was obtained from the Institutional Review Board, Medical Research Center, Hamad Medical Corporation (approval number MRC-01-20-959, dated November 24, 2020). The study was conducted in compliance with national regulations and institutional guidelines for research involving human participants. Identifiable patient data were anonymized to ensure confidentiality.

## Results

The study included 62 patients: 22 (35.5%) male individuals and 40 (64.5%) female individuals. There were 25 (40.3%) Qatari nationals and 37 (59.7%) non-Qatari nationals. Among the patients, 51 (82.3%) had hypertension, 49 (79.0%) had diabetes, and 14 (22.6%) had congestive heart failure. Ischemic heart disease was found in 18 (29.0%) patients, and 17 (27.4%) had a stroke. Peripheral vascular disease was observed in 19 (30.6%) patients, but chronic pulmonary disease was absent in all 62 (100.0%) patients. Thyroid disease was more frequent in female patients (7 (17.5%)) than in male patients (1 (4.5%)), with a total of 8 (12.9%) as shown in Table 1. Chronic kidney disease (CKD) affected 26 (41.9%) patients, with female patients showing a slightly higher frequency.

**Table 1:** Gender wise comparison of clinical characteristics CKD: chronic kidney disease; Clinical Ph: clinical pharmacist; RT: respiratory therapy; PT: physical therapy; OT: occupational therapy; SALT: speech and language therapy; WCNS: wound care nursing service; CDC: Communicable Disease Center; HGH ED: Hamad General Hospital Emergency Department; ICU: intensive care unit; LT: long-term care; QRI: Qatar Rehabilitation Institute

Variables	Male	Female	Total (%)
Nationality			
Qatari	8 (36.4%)	17 (42.5%)	25 (40.3%)
Non-Qatari	14 (63.6%)	23 (57.5%)	37 (59.7%)
Comorbidities			
Hypertension	19 (86.4%)	32 (80.0%)	51 (82.3%)
Diabetes mellitus	18 (81.8%)	31 (77.5%)	49 (79.0%)
Congestive heart failure	5 (22.7%)	9 (22.5%)	14 (22.6%)
Ischemic heart disease	6 (27.3%)	12 (30.0%)	18 (29.0%)
Stroke	8 (36.4%)	9 (22.5%)	17 (27.4%)
Peripheral vascular disease	9 (40.9%)	10 (25.0%)	19 (30.6%)
Thyroid disease	1 (4.5%)	7 (17.5%)	8 (12.9%)
CKD	8 (36.4%)	18 (45.0%)	26 (41.9%)
Dementia	1 (4.5%)	1 (2.5%)	2 (3.2%)
Clinical tests			
First Covid test	4 (18.2%)	3 (7.5%)	7 (11.3%)
Second Covid test	0 (0.0%)	1 (2.5%)	1 (1.6%)
Comprehensive geriatric assessment	22 (100.0%)	40 (100.0%)	62 (100.0%)
Dietitian	22 (100.0%)	40 (100.0%)	62 (100.0%)
Clinical Ph	22 (100.0%)	40 (100.0%)	62 (100.0%)
RT	22 (100.0%)	40 (100.0%)	62 (100.0%)
PT	22 (100.0%)	40 (100.0%)	62 (100.0%)
OT	22 (100.0%)	40 (100.0%)	62 (100.0%)
SALT	22 (100.0%)	40 (100.0%)	62 (100.0%)
WCNS	22 (100.0%)	40 (100.0%)	62 (100.0%)
In-hospital morbidity	2 (9.1%)	2 (5.0%)	4 (6.5%)
Disposition			
CDC	0 (0.0%)	1 (2.5%)	1 (1.6%)
HGH ED	1 (4.5%)	2 (5.0%)	3 (4.8%)
Home	12 (54.6%)	18 (45.0%)	30 (48.4%)
ICU	1 (4.5%)	0 (0.0%)	1 (1.6%)
LT	7 (31.8%)	16 (40.0%)	23 (37.1%)
QRI	1 (4.5%)	3 (7.5%)	4 (6.5%)

All patients received a comprehensive geriatric assessment, and dietitians, clinical pharmacists, respiratory therapists, physical therapists, occupational therapists, speech and language therapists, and wound care nursing staff, regardless of sex or nationality, as shown in Table 2.

**Table 2:** Nationality wise comparison of clinical characteristics CKD: chronic kidney disease; Clinical Ph: clinical pharmacist; RT: respiratory therapy; PT: physical therapy; OT: occupational therapy; SALT: speech and language therapy; WCNS: wound care nursing service; CDC: Communicable Disease Center; HGH ED: Hamad General Hospital Emergency Department; ICU: intensive care unit; LT: long-term care; QRI: Qatar Rehabilitation Institute

Variables	Qatari	Non-Qatari	Total (%)
Comorbidities			
Hypertension	22 (88.0%)	29 (78.4%)	51 (82.3%)
Diabetes mellitus	20 (80.0%)	29 (78.4%)	49 (79.0%)
Congestive heart failure	3 (12.0%)	11 (29.7%)	14 (22.6%)
Ischemic heart disease	4 (16.0%)	14 (37.8%)	18 (29.0%)
Stroke	5 (20.0%)	12 (32.4%)	17 (27.4%)
Peripheral vascular disease	8 (32.0%)	11 (29.7%)	19 (30.6%)
Thyroid disease	4 (16.0%)	4 (10.8%)	8 (12.9%)
CKD	12 (48.0%)	14 (37.8%)	26 (41.9%)
Dementia	0 (0.0%)	2 (5.4%)	2 (3.2%)
Clinical tests			
First Covid test	4 (16.0%)	3 (8.1%)	7 (11.3%)
Second Covid test	0 (0.0%)	1 (2.7%)	1 (1.6%)
Comprehensive geriatric assessment	25 (100.0%)	37 (100.0%)	62 (100.0%)
Dietitian	25 (100.0%)	37 (100.0%)	62(100.0%)
Clinical Ph	25 (100.0%)	37 (100.0%)	62 (100.0%)
RT	25 (100.0%)	37 (100.0%)	62 (100.0%)
PT	25 (100.0%)	37 (100.0%)	62 (100.0%)
OT	25 (100.0%)	37 (100.0%)	62 (100.0%)
SALT	25 (100.0%)	37 (100.0%)	62 (100.0%)
WCNS	25 (100.0%)	37 (100.0%)	62 (100.0%)
In-hospital morbidity	2 (8.0%)	2 (5.4%)	4 (6.5%)
Disposition			
CDC	0 (0.0%)	1 (2.7%)	1 (1.6%)
HGH ED	2 (8.0%)	1 (2.7%)	3 (4.8%)
Home	8 (32.0%)	22 (59.4%)	30 (48.4%)
ICU	0 (0.0%)	1 (2.7%)	1 (1.6%)
LT	15 (60.0%)	8 (21.6%)	23 (37.1%)
QRI	0 (0.0%)	4 (10.8%)	4 (6.5%)

In-hospital morbidity was observed in 4 (6.5%) patients with no significant differences in sex or nationality. Throughout the cohort, 0 (0.0%) patients died in the hospital. In terms of post-hospitalization disposition, most Qatari patients were transferred to long-term care (15 (60.0%)), whereas non-Qatari patients were discharged home (14 (37.8%)).

The presence of comorbidities varied slightly by nationality, with Qatari patients showing a higher prevalence of hypertension (22 (88.0%) vs. 29 (78.4%)), while non-Qatari patients had a higher prevalence of ischemic heart disease (14 (37.8%) vs. 4 (16.0%)) and congestive heart failure (11 (29.7%) vs. 3 (12.0%)). These findings suggest that certain comorbidities were more prevalent in specific nationality groups, yet healthcare interventions, including comprehensive geriatric assessments and multidisciplinary care, were applied consistently across all 62 (100.0%) patients.

## Discussion

The findings of this study highlight the efficacy of a multidisciplinary strategy in managing older adults with several comorbidities during the COVID-19 pandemic. Despite the high prevalence of chronic conditions such as hypertension, diabetes, and ischemic heart disease among quarantined patients, the absence of in-hospital mortality and low morbidity rate demonstrate the efficacy of Rumailah Hospital's comprehensive care protocols. The literature suggests the benefits of multidisciplinary treatment in lowering mortality and improving outcomes in patients with severe COVID-19, particularly in older populations with complicated health profiles [2,5,7,8]. A review by McCarthy et al. found that older adults with COVID-19 showed significant improvements in their ability to function after receiving multidisciplinary inpatient rehabilitation, with a Standardized Mean Difference (SMD) of 1.46 (95% CI 0.94 to 1.98) [7]. This evidence supports our study's findings that a multidisciplinary approach can effectively manage complex cases and improve patient outcomes. In a similar way, O'Brien et al. showed that a combined approach with respiratory, critical care, and mental health services helped recognize and meet the greater physical and mental health needs of patients after COVID-19 pneumonia, with 48% needing follow-up care for a few months to a longer time [8].

The standardized implementation of care across genders (22 (35.5%) male patients, 40 (64.5%) female patients) and nations (25 (40.3%) Qatari, 37 (59.7%) non-Qatari) indicates that standardized treatment methods may successfully eliminate inequities in healthcare delivery, even in a heterogeneous patient population. This equity in care delivery resonates with Zhu et al.’s emphasis on integrated care models for geriatric health during pandemics [9]. However, the higher proportion of long-term care placement among Qatari patients (15 (60.0%) vs. 14 (37.8%) non-Qatari patients discharged home) may reflect varying post-discharge care needs influenced by sociocultural factors, which warrant further investigation. A study by Hammad et al. on dementia caregiving in Arab and Muslim communities, including Qatar, revealed that sociocultural and religious influences, such as family roles and religious attitudes, significantly shape caregiving experiences [10]. These factors may contribute to the observed differences in post-discharge outcomes. Additionally, Lebrasseur et al. highlighted that loneliness, ageism, and limited digital access significantly affect elderly patients’ outcomes, suggesting these factors may influence post-discharge care disparities [11].

Psychological interventions played a crucial role in our multidisciplinary approach, likely reducing morbidity by addressing mental health needs. Haddad et al.’s study on quarantine-related stressors and eating behaviors suggests that such interventions are essential during quarantine [4]. Furthermore, a cross-sectional study in Qatar by Reagu et al. reported that 37.4% of individuals in institutional quarantine experienced depressive symptoms, and 25.9% had anxiety symptoms, highlighting the psychological burden of quarantine [12]. However, Ouanes et al. found that elderly individuals under COVID-19 quarantine in Qatar did not develop significant psychological distress, possibly due to high resilience and effective coping strategies, including religious coping [13]. These findings underscore the importance of integrated, patient-centered care systems that address both medical and psychological requirements, especially in vulnerable populations with multiple morbidities during public health emergencies [5]. Prendki et al. described that the atypical manifestations of COVID-19 in older patients can be delirium or neurological involvement, which might result in an incorrect diagnosis and a higher rate of mortality, which is even more evidence of the importance of thorough preliminary evaluations [14].

Our study and others clearly indicate that the management of patients with multiple morbidities in a quarantine environment has some specific issues. In a retrospective case series by Al Hamad et al. on a COVID-19 outbreak within a long-term care facility in Qatar, 24 patients were involved, and seven of them were elderly, and they reported three deaths, highlighting the high-risk population as well as the importance of infection control procedures [15]. The success of our study in containing the spread and effectively managing the patients could be credited to the proactive intervention and multi-hyphen care protocols by the multidisciplinary team. In the same way, Montani et al. recommended multidisciplinary care when managing post-acute COVID-19 syndrome and highlighted the necessity to eliminate the boundaries between medical subspecialties to provide the best support possible [16].

Context regarding the increased risks of poor outcomes of COVID-19 among elderly patients with comorbidities is extensively reported. As the Centers for Disease Control and Prevention (CDC) has observed, when conditions such as hypertension and diabetes are present, the risk of severe illness is dramatically raised [17]. An additional piece of evidence to prove this point is a systematic review by Perefki et al., which emphasizes the importance of the fact that comorbidities increase the risk of mortality in elderly COVID-19 patients significantly [18]. The multidisciplinary nature of our study helped us manage these risks because we offered specialized care to reduce the effects of these comorbidities.

It is also important to address the long-term effects and the follow-up care. In a rapid review conducted by Lebrasseur et al., ongoing support was discussed as the means to leverage social isolation and mental health challenges among older adults during the pandemic [11]. The recommendation on comprehensive geriatric assessment and rehabilitation fits the scope of our study, as we want to ensure that patients are supported in a holistic fashion beyond the acute period.

The study is subject to several important limitations that compromise its scientific rigor and overall impact. The small sample size and single-site setting restrict the generalizability of the findings to larger and more diverse populations, as well as to care settings beyond quarantine facilities. Additionally, the retrospective design relies on existing data, which may be incomplete, inconsistent, or subject to selection and information biases. The exclusive use of descriptive statistics, without inferential analyses (e.g., p-values, confidence intervals, or regression models), limits the ability to draw robust conclusions about the effectiveness of the multidisciplinary approach and precludes causal inferences. The focus on in-hospital prognosis and immediate discharge outcomes also provides limited insight into long-term patient outcomes and quality of life. Furthermore, the use of electronic health records may introduce errors or data gaps, and external factors such as national healthcare policies and evolving treatment protocols could have influenced the results. We acknowledge that previous reviewer feedback-such as recommendations to include statistical indicators and enhance methodological transparency, was not fully incorporated in earlier revisions, which may have contributed to an overly optimistic portrayal of the findings. Given these constraints, the results should be interpreted with considerable caution, and there is a clear need for larger, multicenter prospective studies with comparative designs and rigorous statistical methods to validate and extend these observations across different care environments.

## Conclusions

The findings from this retrospective study indicate that the multidisciplinary intervention was associated with low in-hospital morbidity and no in-hospital mortality among elderly patients with multiple comorbidities during the COVID-19 pandemic. Differences in post-discharge disposition were observed, with a higher proportion of Qatari patients transferred to long-term care compared to non-Qatari patients, who were more frequently discharged home. These descriptive results suggest potential benefits of comprehensive, multidisciplinary care in managing adverse outcomes in vulnerable populations during quarantine, though causal inferences cannot be drawn due to the study's non-comparative design. Future research should explore sociocultural and healthcare system factors contributing to post-discharge disparities, such as long-term care placement, and assess the scalability of multidisciplinary models through larger, prospective, and comparative studies in diverse quarantine settings.
